# Associação entre Infecção por Heicobacter Pylori e Hipertensão Arterial Sistêmica: Metanálise

**DOI:** 10.36660/abc.20200186

**Published:** 2021-09-03

**Authors:** Mengyun Huang, Lijun Zhu, Yuelong Jin, Zhengmei Fang, Yan Chen, Yingshui Yao

**Affiliations:** 1 Wannan Medical College/ Institute of Chronic Disease Prevention and Control School of Public Health Department of Epidemiology and Biostatistics Wuhu China Department of Epidemiology and Biostatistics, School of Public Health, Wannan Medical College/ Institute of Chronic Disease Prevention and Control, Wuhu – China; 2 Anhui College of Traditional Chinese Medicine Department of Medicine Wuhu China Department of Medicine, Anhui College of Traditional Chinese Medicine, Wuhu – China

**Keywords:** Hipertensão, Pressão ArterialPressão Arterial, Microbiota, Infecção, Epidemiologia, Helicobacter pylori, Fatores de Risco, Endotélio Vascular, Doenças Cardiovasculares, Metanálise

## Abstract

**Fundamento:**

Estudos epidemiológicos recentes demonstraram que alterações na microbiota e seus metabólitos estão associadas à hipertensão arterial sistêmica. A Helicobacter pylori (H. pylori) é um dos patógenos bacterianos mais comuns, e a possível associação entre a infecção por H. pylori e a hipertensão é controversa.

**Objetivos:**

Este estudo teve o objetivo de esclarecer a associação entre eles e proporcionar uma nova base teórica para detectar a patogênese da hipertensão.

**Métodos:**

Foram selecionados estudos caso-controle e transversais sobre a associação entre H. pylori e hipertensão, publicados de 1996 a 2019 indexados nos bancos de dados PubMed, Google Scholar, Chinese Wan Fang Data, e Chinese National Knowledge Infrastructure (CNKI). As razões de chance (RC) combinadas e o intervalo de confiança (IC) 95% foram estimados. O I² foi realizado para avaliar a heterogeneidade estatística. O viés de publicação foi avaliado utilizando-se os testes de Beggs e de Egger. Os dados extraídos foram analisados no software Stata 12.0. A significância estatística foi definida com um p-valor < 0,05.

**Resultados:**

Foram cadastrados 17 estudos envolvendo 6376 casos de hipertensão e 10850 controles. A taxa de infecção por H. pylori em pacientes hipertensos e em controles foi de 64,9% e 56,3%, respectivamente. Foi demonstrada uma associação significativamente positiva entre a infecção por H. pylori e a hipertensão, com uma RC global de 2,07 (IC 95%: 1,46–2,94; p < 0,05). A análise de subgrupos revelou que a prevalência de infecção por H. pylori foi associada à hipertensão na região da Ásia e no grupo de caso-controle, as RC (IC 95%) foram 2,26 (1,51-3,38) e 2,53 (1,72-3,72), respectivamente. Depois de estratificar por métodos de detecção, ainda existiam diferenças entre os subgrupos (todos p < 0,05).

**Conclusão:**

Esta metanálise indicou que a infecção por H. pylori está associada positivamente à hipertensão.

## Introdução

A hipertensão arterial sistêmica, também conhecida como pressão arterial alta ou elevada, é uma condição em que os vasos sanguíneos aumentam a pressão constantemente. De acordo com a Organização Mundial de Saúde (OMS), aproximadamente 1,13 bilhões de pessoa em todo o mundo têm hipertensão, e dois-terços delas vivem em países com renda baixa ou média. ^1^ Na China, cerca de 270 milhões de pessoas têm hipertensão, e sua prevalência é mais alta no Norte e mais baixa no Sul. ^11^ Como um problema de saúde pública global, a hipertensão contribui para a carga de doenças cardíacas, acidente vascular cerebral, insuficiência renal, e outras doenças. ^3^ Ela é considerada um distúrbio causal complexo, por ser influenciada pela interação entre vários fatores, tais como má alimentação, uso indevido de álcool, sedentarismo, tabagismo e fatores genéticos. ^4^ Recentemente, estudos em seres humanos e animais demonstraram que alterações na microbiota e seus metabólitos estão associados à hipertensão arterial sistêmica. ^5^^,^^6^

A H. pylori é um dos patógenos bacterianos mais comuns, e existe no piloro do estômago humano. ^7^ A prevalência da H. pylori, em países específicos varia de 18,9% na Suíça a 87,7% na Nigéria. ^8^ Ela já infectou mais da metade da população mundial. ^9^ A infecção por H. pylori causa inflamação crônica ativa com um recrutamento contínuo de neutrófilos para a mucosa gástrica inflamada. ^10^ Além disso, outro estudo demonstrou que a microbiota intestinal facilita a disfunção vascular induzida por AngII e a hipertensão, causando a infiltração e a inflamação de células imunes vasculares. ^11^ Um estudo recente relatou que a soropositividade para H. pylori está intimamente relacionada a aterosclerose, e a infecção por H. pylori pode contribuir para o desenvolvimento de doenças cardiovasculares. ^12^ A pesquisa em animais demonstrou que a coinfecção de Chlamydia pneumoniae e H. pylori levou à disfunção endotelial vascular e aumentou a expressão de VCAM-1 em ratos. ^13^ Esses achados destacam o papel importante desempenhado pela H. pylori na regulação da disfunção endotelial e do sistema AngIII, e a possibilidade de a H. pylori estar envolvida no desenvolvimento da hipertensão.

Um estudo transversal envolvendo 5.246 participantes detectou uma associação positiva entre a infecção por H. pylori e a hipertensão após o ajuste de possíveis fatores de confusão. ^14^ Inversamente, o status de H. pylori não foi significativamente diferente em pacientes com graus de hipertensão diferentes. ^15^ Considerando a introdução acima e a diversidade de resultados sobre a infecção por H. pylori e hipertensão, a necessidade de ser realizar um estudo para determinar a relação entre infecção por H. pylori e hipertensão fica muito clara. Portanto, para investigar em mais detalhes o possível papel da infecção por H. pylori na hipertensão, conduzimos uma metanálise para proporcionar uma base para a intervenção na hipertensão.

## Métodos

### Coleta da literatura

Todos os trabalhos sobre a relação entre H. pylori e hipertensão publicados entre 1996 e 2019 foram selecionados para esta metanálise. Foram pesquisados artigos cujos títulos e/ou resumos continham as expressões “H. pylori” ou “Helicobacter pylori” e “hipertensão” ou “pressão arterial alta”, publicados em chinês nos bancos de dados Chinese Wanfang Data Knowledge Service Platform e Chinese National Knowledge Infrastructure (CNKI), e em inglês nos bancos de dados PubMed e Google Scholar. Por último, as referências foram filtradas novamente para evitar omissão, no processo de leitura de triagem dos artigos.

### Critérios de inclusão e de exclusão

Todos os estudos que foram identificados pela pesquisa de literatura foram selecionados de acordo com os seguintes elementos essenciais: pacientes (indivíduos que foram diagnosticados de acordo com o padrão de diagnóstico de hipertensão arterial sistêmica); exposição (infecção por H. pylori); comparador (normotensão); resultado (associação entre infecção por H. pylori e hipertensão); e desenho do estudo (estudo transversal ou caso-controle). Foram excluídos estudos ecológicos e de coorte, os que não agruparam por hipertensão e normotensão, aqueles em que o número de pacientes com H. pylori em cada grupo não possa ser determinado, ou devido a possíveis erros. Nos casos em que o mesmo estudo apareceu em bancos de dados diferentes ou em que a população do estudo coincidiu, apenas o maior foi selecionado.

### Extração de dados e avaliação de qualidade no processo

Em conformidade com o objetivo deste estudo, dois pesquisadores independentes selecionaram títulos e/ou resumos para serem incluídos nos artigos, e chegou-se a um consenso final pela avaliação de um terceiro especialista. Os estudos necessários foram lidos na íntegra, e as seguintes informações e características foram registradas: nome do primeiro autor, ano de publicação, país, tipo de estudo, média de idade, número de participantes, e testes de infecção por H. pylori.

## Análise estatística

Todas as análises estatísticas foram realizadas utilizando-se o software Stata 12.0. As razões de chance (RC) combinadas, com os intervalos de confiança (IC) de 95% correspondentes, foram consideradas tamanho de efeito para todos os estudos qualificados. Dois métodos (o teste de Cochran e a estatística I²) firam usados para avaliar a heterogeneidade estatística entre os dados resumidos: o p<0,05 foi considerado estatisticamente significativos quanto à heterogeneidade, e a estatística I² sugere heterogeneidade significativa com um valor >50%. ^16^ Um modelo de efeito randômico foi usado para calcular a estimativa de tamanho de efeito geral para esta metanálise. Para examinar as fontes de heterogeneidade entre os estudos qualificados, foram realizadas análises de subgrupos de acordo com características diferentes, tais como desenho do estudo (estudos transversais ou caso-controle), país do estudo (asiáticos ou ocidentais), e testes para infecção por H. pylori (teste respiratório com ureia marcada, testes sorológicos ou outros). Os testes respiratórios com ureia marcada (UBT) incluíram o 13C-UBT e o 14C-UBT, os testes sorológicos incluíram o método Colloidal gold e o ELISA. Outros testes incluíram a Coloração de Giemsa e os casos em que as informações não estavam disponíveis (N/D). A análise de sensibilidade foi realizada para avaliar os efeitos do estudo específico na estimativa de efeitos resumidos e na estabilidade dos resultados. Os testes de regressão de Begg e Egger foram usados para avaliar o viés de publicação. Foi realizado o método de metarregressão para dados de medição, tais como, tamanho da amostra, média de idade e razão de sexos. Um p valor bicaudal < 0,05 foi definido como estatisticamente significativo.

## Resultados

### Características básicas dos artigos ^17^^-^^33^

**Figura 1:**
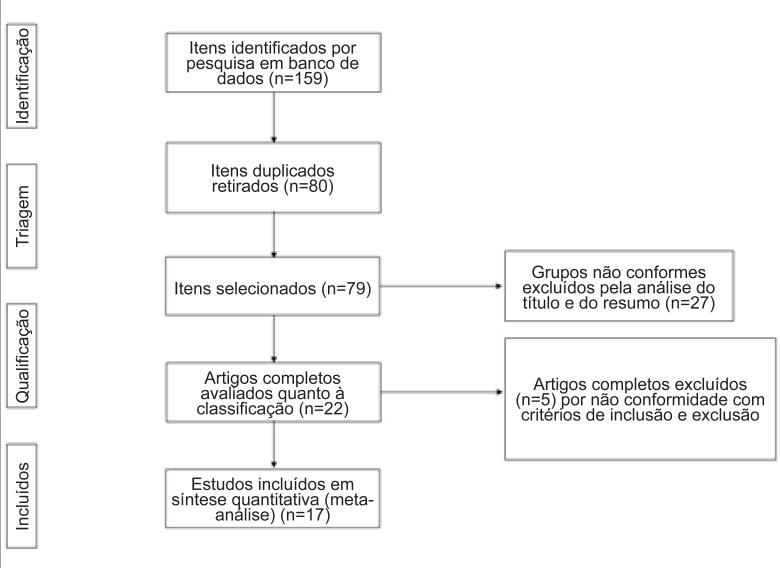
Fluxograma dos estudos incluídos na meta-análise.

Foi apresentado um fluxograma de estudos para revisão sistemática na Figura 1 . Desses estudos, foram excluídos 80 estudos duplicados, e, em seguida, com base nos títulos e resumos dos artigos restantes, foram excluídos 57 estudos. Ao final, 17 publicações foram selecionadas após realizar o exame do texto dessas 22 publicações na íntegra, conforme especificado no fluxograma.

No total, 6 estudos transversais e 11 estudos caso-controle foram selecionados na metanálise, no período de 1996 a 2019. Entre os 17.226 participantes, a prevalência de H. pylori em 6.376 pacientes hipertensos foi de 64,9, e em 10.850 normotensos foi de 56,3%. As principais características dos estudos incluídos nesta revisão foram apresentadas na Tabela 1 . ^17^^-^^33^

**Tabela 1:** Características básicas dos dezessete artigos incluídos

Estudos	Ano	País	Constatação da H. pylori	Hipertensão (H. pylori + / -)	Normotensos (H. pylori + / -)	Média de idade	Desenho do estudo	Razão de sexos	limiar p
Yang ^17^	2016	China #	14C-UBT	78(66/12)	78(37/41)	N/D	Estudo caso-controle	0,79	0,05
Liu ^18^	2015	China #	14C-UBT	150(125/25)	150(71/79)	63,60	Estudo caso-controle	1,04	0,05
Huai ^19^	2014	China #	13C-UBT	535(338/197)	1051(232/819)	68,30	Estudo transversal	2,68	0,05
Ma ^20^	2015	China #	Coloração de Giemsa	112(42/70)	170(53/117)	N/D	Estudo caso-controle	N/D	0,05
Chu ^21^	2014	China #	IgG Colloidal gold	150(67/83)	50(14/36)	60,85	Estudo caso-controle	1,41	0,05
Hu ^22^	2017	China #	N/D	176(82/94)	568(224/344)	52,53	Estudo transversal	1,23	0,05
Lip ^23^	1996	Reino Unido $	ELISA≥8 unidades/ml).	124(106/18)	38(25/13)	53,06	Estudo caso-controle	1,03	0,05
Liu ^24^	2007	China #	ELISA	488(189/299)	942(317/625)	46,56	Estudo transversal	0,71	0,05
Sun ^25^	2018	China #	14C-UBT	90(76/14)	65(34/31)	44,74	Estudo caso-controle	1,12	0,05
Li ^26^	1999	China #	IgG ELISA	42(16/26)	60(18/42)	55,41	Estudo caso-controle	1,62	0,05
Zhao ^27^	2019	China #	14C-UBT	102(43/59)	102(18/84)	47,85	Estudo caso-controle	0,94	0,05
Migneco ^28^	2003	Itália $	13C-UBT	72(40/32)	70(35/35)	52,51	Estudo caso-controle	0,95	0,05
Vahdat ^29^	2013	Irã #	ELISA >30 RU/ml	459(316/143)	1295(764/531)	40,79	Estudo transversal	0,97	0,05
Zheng ^30^	2014	China #	Coloração de Giemsa	112(42/70)	170(53/117)	52,19	Estudo caso-controle	1,29	0,05
Sung ^31^	2003	Coreia #	ELISA≥6 U/mL	2838(1964/874)	5509(3818/1691)	48,06	Estudo transversal	1,36	0,05
Sotuneh ^32^	2014	Itália $	ELISA >20 ur/ml	808(606/202)	492(385/107)	69,23	Estudo transversal	N/D	0,05
S.VSM ^33^	2012	Índia #	IgG ELISA > 40 ur/ml	40(18/22)	40(9/31)	44,09	Estudo caso-controle	1,05	0,05

UBT: teste respiratório com ureia marcada; ELISA: ensaio de imunoabsorção enzimática; N/D: não disponível;

* Razão de sexos: masculino/feminino

# países asiáticos

$ países ocidentais.

### Resultados da metanálise

**Figura 2:**
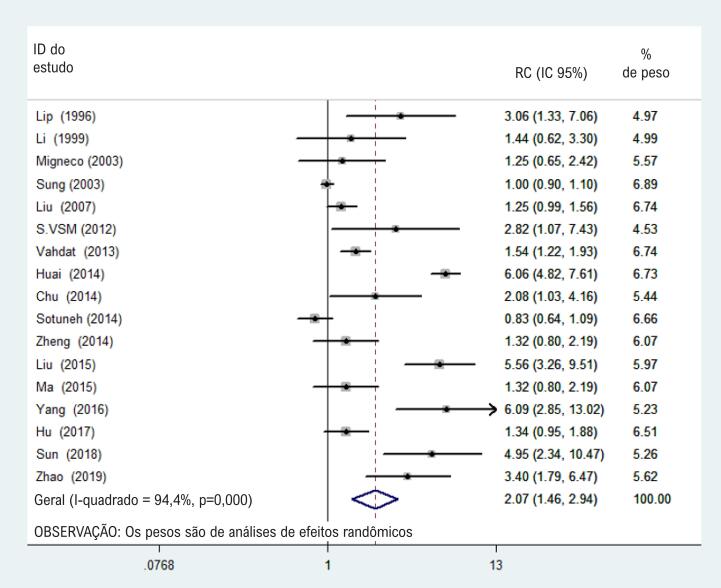
Gráfico de floresta da associação entre infecção por H. pylori infecção e hipertensão.

Com base no modelo de efeitos randômicos da Figura 2 , a estimativa geral das RC combinadas (IC 95%) de H. pylori e hipertensão foi 2,07 (1,46–2,94), o que era estatisticamente significativo (p < 0,001).

### Análise de subgrupos por região, desenho de estudo, e método de teste

Para a análise por subgrupo por região (em países asiáticos, 5372 pacientes hipertensos e 10250 normotensos, e em países ocidentais, 1004 pacientes hipertensos e 600 normotensos), a infecção por H. pylori foi associada ao risco de hipertensão em países asiáticos (RC 2,26, IC 95% 1,51-3,38; I^2^ = 95,1%, p < 0,05), e não houve diferenças significativas nos países ocidentais.

**Figura 3:**
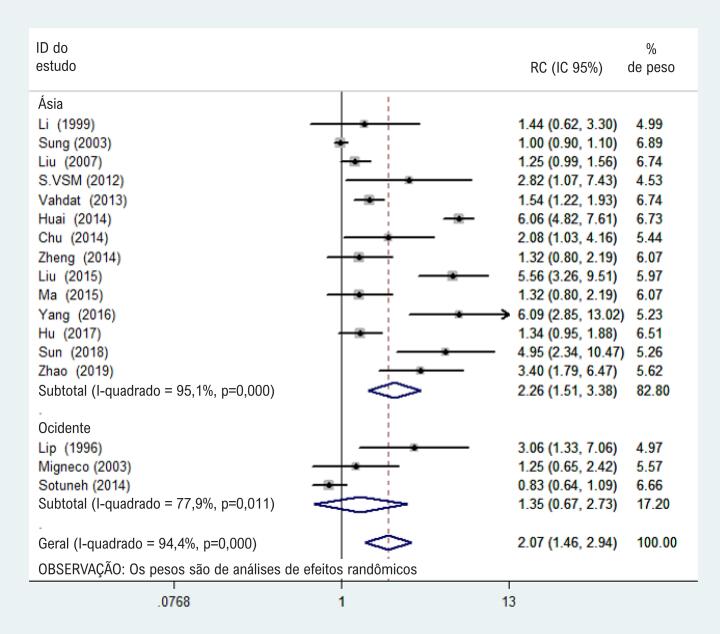
Gráfico de floresta de análises de subgrupos comparando a Ásia ao Ocidente.

Na análise por subgrupo por desenho do estudo, a RC combinada para infecção por H. pylori e hipertensão foi de 2,53 (IC 95% 1,72-3,72; I^2^ = 72,7%, p < 0,05) nos estudos caso-controle. Não se observaram diferenças estatísticas nos estudos transversais (Figuras 3 e 4 ).

**Figura 4:**
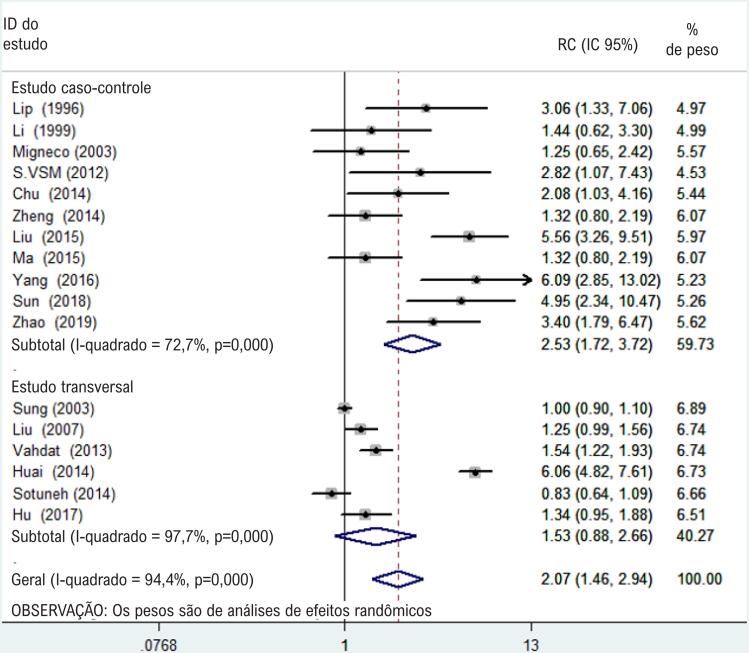
Gráfico de floresta de análises de subgrupos estudos caso-controle a estudos transversais.

**Figura 5:**
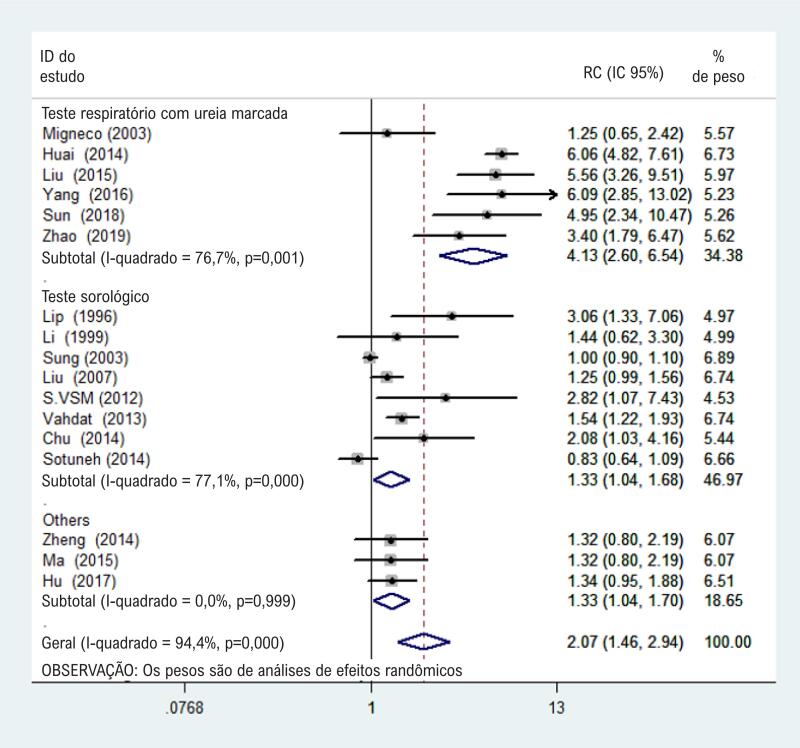
Gráfico de floresta de análises de subgrupos comparando vários métodos de diagnóstico.

Na análise por subgrupo que utilizou o UBT para testar quanto à infecção por H. pylori, esta foi associada a um risco mais alto de hipertensão (RC 4,13, IC 95% 2,60-6,54; I^2^ = 76,7%, p < 0,05). A infecção por H. pylori também foi associada a um risco mais alto de hipertensão na análise por subgrupo que utilizou testes sorológicos (RC 1,33, IC 95% 1,04-1,68; I^2^ = 77,1%, p < 0,05) ( Figura 5 ).

### Viés de publicação

**Figura 6:**
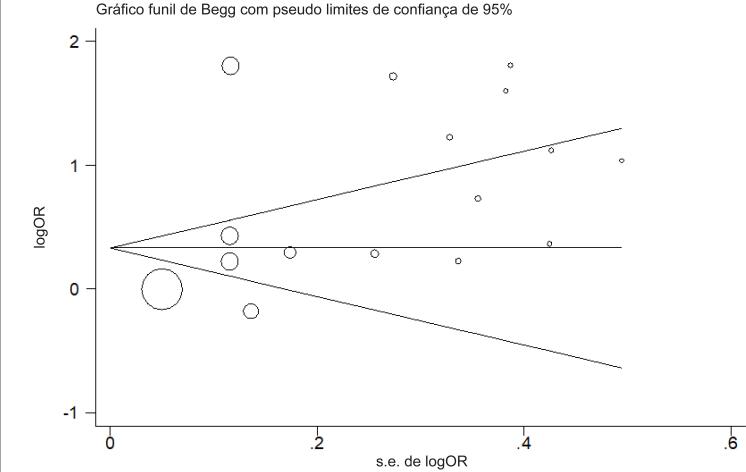
Gráfico de funil com pseudolimite de confiança de 95%.

O teste de Begg foi utilizado para avaliar o viés de publicação com o gráfico de funil ( Figura 6 ). Houve uma assimetria no gráfico de funil para os estudos selecionados. O viés de publicação significativo também foi identificado no teste de regressão de Egger (p = 0,047).

### Análise de sensibilidade

**Figura 7:**
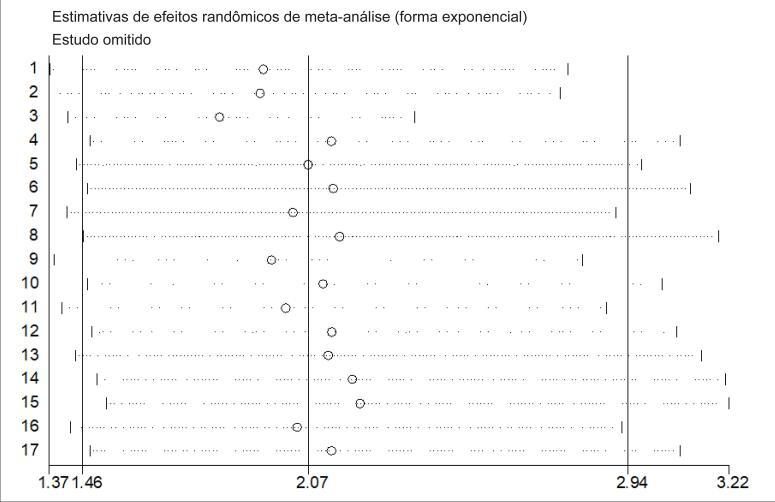
Análise de sensibilidade para associação entre H. pylori e hipertensão.

Realizamos uma análise de sensibilidade para testar as fontes de heterogeneidade e avaliamos a estabilidade dos resultados. Os resultados da análise de sensibilidade demonstraram que nenhum estudo específico teve influência extrema na razão de chances combinada ( Figura 7 ).

### Metarregressão

Estudos específicos sem média de dados (n = 2) ou sem dados de sexo masculino ou feminino (n = 2) foram excluídos. Os resultados de metarregressões univariadas mostram a ausência de efeitos significativos de tamanho de amostra (p = 0,181), média de idade (p = 0,542), razão de sexos (p = 0,367) na associação entre a infecção por H. pylori e o risco de hipertensão nos estudos (Material Suplementar: Figuras suplementares 1-3).

## Discussão

Até onde sabemos, este é o primeiro estudo de revisão sistemática que demonstra a relação entre a infecção por H. pylori e a hipertensão. Os achados demonstraram que a prevalência da infecção por H. pylori foi associada positivamente à hipertensão, e esse resultado foi consistente no grupo de caso-controle na análise por subgrupo. Mendel et al. ^34^ foram os primeiros a levantar a hipótese da relação entre a infecção por H. pylori e as doenças cardíacas coronárias. Depois disso, alguns acadêmicos realizaram pesquisas sobre a infecção por H. pylori em doenças cardiovasculares. ^35^

Um estudo de coorte prévio demonstrou que indivíduos infectados por H. pylori, com diagnóstico positivo de gastrite ativa crônica, tinham o risco 29% mais alto de desenvolver hipertensão durante o período de acompanhamento. ^36^ Um estudo chinês indicou que a infecção por H. pylori foi independentemente associada a PAD mais alta, mas não PAS mais alta depois de se ajustar as covariáveis. ^14^ Entretanto, vários estudos demonstraram que a infecção por H. pylori não influenciou a pressão arterial. ^37^^,^^38^ Obviamente, não podemos concluir que a infecção por H. pylori facilita a hipertensão. Entretanto, nossa metanálise agora sustenta a associação entre a infecção por H. pylori e a hipertensão.

A prevalência da infecção por H. pylori varia notadamente em vários países e regiões. As infecções por H. pylori em países asiáticos eram comuns e distribuídas por uma grande extensão. A taxa média de infecção na China foi de 58,07%, com 50% na faixa etária entre 10 e 20 anos. ^39^ A investigação epidemiológica demonstrou que a cepa positiva para o gene A associado a citocina (gene cagA) de várias áreas geográficas apresenta clara diferenciação filogeológica. Mais de 90% dos isolados de H. pylori de países da Ásia Ocidental, tais como a China e o Japão, contêm proteína cagA, enquanto apenas 60% a 70% dos isolados de H. pylori em países ocidentais, tais como os Estados Unidos, contêm a proteína cagA. ^40^ Além disso, Migneco A et al. ^28^ identificaram que apenas entre os pacientes com resultado positivo alto para cagA a PAD diminuiu mais claramente depois de o H. pylori ter sido erradicado, e isso pode estar ligado ao relacionamento molecular entre o antígeno cagA da H. pylori e alguns peptídeos expressos por células endoteliais e células de músculo liso. É notável que a RC da hipertensão em populações asiáticas com infecção por H. pylori foi 2,26 vezes maiores do que as de sujeitos normotensos em nosso estudo. Esses resultados sugerem que a origem étnica pode ter um possível impacto na relação entre a infecção por H. pylori e a hipertensão.

Outro problema para se determinar a infecção por H. pylori e hipertensão é o método utilizado para testar a infecção por H. pylori. Embora os testes sorológicos sejam uma forma de triagem comum, eles não garantem a identificação da infecção por H. pylori ativa. ^41^ A precisão do diagnóstico utilizando-se o UBT para se detectar a infecção por H. pylori na população asiática, especialmente o 13C-UBT, que tinha uma precisão diagnóstica impressionante, com uma sensibilidade de 97% e uma especificidade de 96%. ^42^ Em nossa análise por subgrupo, o risco de hipertensão era mais alto quando a infecção por H. pylori foi determinada utilizando-se um UBT, em comparação com outros testes.

Os mecanismos que ligam a infecção por H. pylori à hipertensão ainda não estão claros. Há várias hipóteses que sustentam a relação, e uma das mais plausíveis é a dos níveis de citocinas inflamatórias. Epstein et al. ^43^ indicaram que a inflamação crônica causada pela infecção por H. pylori pode levar à aterosclerose avançada, consequentemente. Além disso, um estudo prospectivo demonstrou que a H. pylori tinha uma associação positiva com LDL alto e HDL baixo. ^44^ Os lipídeos, como parte integral da membrana celular, têm um papel importante no desenvolvimento da hipertensão. ^45^ Especula-se que a infecção por H. pylori leva ao metabolismo anormal de LDL-C, HDL-C e TC, o que, por sua vez, resulta em hipertensão. A infecção por H. pylori destrói a tolerância imune e causa reação autoimune, que pode fazer parte da patogênese da hipertensão. ^46^^,^^47^ Identificou-se que a idade é um preditor independente da infecção por H. pylori, e há uma tendência crescente de prevalência com a idade, apesar de não existir diferença de prevalência entre os sexos. ^48^ Além disso, os homens têm duas vezes mais probabilidade de desenvolver doenças cardiovasculares que as mulheres, pelo menos abaixo dos 60 anos. ^49^ Entretanto, nosso estudo mostra a ausência de efeitos significativos de idade e sexo na associação entre a infecção por H. pylori e o risco de hipertensão. É possível que diferenças étnicas, avaliação da infecção por H. pylori, tamanho de amostras, e possíveis fatores de confusão contribuam para a existência de discrepâncias.

### Limitações

Há várias limitações em nosso estudo. Primeiramente, todos os estudos incluídos foram estudos observacionais, o que dificultou a estimativa de uma associação causal. Em segundo lugar, foi identificada uma heterogeneidade nas metanálises e não foi possível ajustar variáveis com o potencial de serem fatores de confusão por outras informações não acessíveis. Em terceiro lugar, mais estudos originais foram realizados na população asiática, e houve um viés de publicação na metanálise.

## Conclusões

Em conclusão, nossos resultados indicaram que a infecção por H. pylori está associada positivamente à hipertensão. As estratégias de prevenção da infecção por H. pylori e a erradicação da H. pylori podem ter um efeito significativo na prevenção e no tratamento da hipertensão, e devem ser avaliadas em mais detalhes.
