# Supplementary material for: Associação entre Infecção por Heicobacter Pylori e Hipertensão Arterial Sistêmica: Metanálise
Source: Arq Bras Cardiol. 2021 Sep 3;117(4):626–36. [Article in Portuguese] doi: 10.36660/abc.20200186 (PMC8528373; doi:10.36660/abc.20200186)
Supplement: Translation

# Translation: Association between Helicobacter Pylori Infection and Systemic Arterial Hypertension: A Meta-Analysis

**Authors:** Mengyun Huang, Lijun Zhu, Yuelong Jin, Zhengmei Fang, Yan Chen, Yingshui Yao

**Affiliations:** 1 Wuhu China Department of Epidemiology and Biostatistics, School of Public Health, Wannan Medical College/ Institute of Chronic Disease Prevention and Control, Wuhu – China; 2 Wuhu China Department of Medicine, Anhui College of Traditional Chinese Medicine, Wuhu – China

**Keywords:** Hypertension, Blood Pressure, Microbiota, Infection, Epidemiology, Helicobacter Pylori, Risk Factors, Endothelium Vascular, Cardiovascular Diseases, Meta-Analysis

## Abstract

**Background:**

Recent epidemiological studies have shown that alterations in microbiota and its metabolites are associated with systemic arterial hypertension. Helicobacter pylori (H. pylori) is one of the most common bacterial pathogens, and the potential association between H. pylori infection and hypertension are controversial.

**Objective:**

This study aimed to clarify their association and provide a new theoretical basis for uncovering the pathogenesis of hypertension.

**Methods:**

Case-control and cross-sectional studies on the association between H. pylori and hypertension published from 1996 to 2019 indexed in PubMed, Google Scholar, Chinese Wan Fang Data, and Chinese National Knowledge Infrastructure (CNKI). The pooled odds ratios (OR) and 95% confidence interval (CI) were estimated. I2 was performed to evaluate the statistical heterogeneity. Publication bias was evaluated using Begg’s and Egger’s test. The extracted data was analyzed in Stata 12.0. Statistical significance was defined as p-value < 0.05.

**Results:**

A total of 17 studies involving 6,376 cases of hypertension and 10,850 controls were enrolled. H. pylori infection rate in hypertension patients and controls were 64.9% and 56.3%, respectively. A significantly positive association was shown between H. pylori infection and hypertension with an overall OR of 2.07 (95% CI: 1.46–2.94; p < 0.05). Subgroup analysis revealed that the prevalence of H. pylori infection was associated with hypertension in the region of Asia and the case-control group, ORs (95% CI) were 2.26 (1.51-3.38) and 2.53 (1.72-3.72), respectively. After stratifying by detection methods, differences still existed in subgroups (all p < 0.05).

**Conclusion:**

This meta-analysis indicated that H. pylori infection is positively associated with hypertension.

## Introduction

Systemic arterial hypertension, also known as high or raised blood pressure, is a condition in which the blood vessels have a persistently high pressure. According to the World Health Organization (WHO), an estimated 1.13 billion people worldwide have hypertension, with two-thirds living in low- and middle-income countries. ^1^ In China, about 270 million people have hypertension, and its prevalence is highest in the North and lowest in the South. ^2^ As a global public health problem, hypertension contributes to the burden of heart disease, stroke, and kidney failure, among other diseases. ^3^ It is considered a complex causal disorder, which be influenced by the interaction among many factors, such as unhealthy diet, harmful use of alcohol, physical inactivity, tobacco use, and genetic factors. ^4^ Recently, studies in humans and animals have shown that alterations in microbiota and its metabolites are associated with hypertension. ^5^^,^^6^

H. pylori is one of the most common bacterial pathogens and exists in the pylorus of the human stomach. ^7^ The prevalence of H. pylori in individual countries varies from 18.9% in Switzerland to 87.7% in Nigeria ^8^ and has infected more than half of the global population. ^9^ H. pylori infection causes active chronic inflammation with a continuous recruitment of neutrophils to the inflamed gastric mucosa. ^10^ Moreover, another study showed that abnormal gut microbiota facilitate AngII-induced vascular dysfunction and hypertension by driving vascular immune cell infiltration and inflammation. ^11^ A recent study has reported that H. pylori seropositivity is closely related to arteriosclerosis, and H. pylori infection may contribute to the development of cardiovascular disease. ^12^ Animal research has revealed that co-infection with Chlamydia pneumoniae and H. pylori result in vascular endothelial dysfunction and enhanced VCAM-1 expression in mice. ^13^ These findings highlight the important role of H. pylori in regulating endothelial dysfunction and the AngII system, and H. pylori infection may be involved in the development of hypertension.

A cross-sectional study involving 5,246 participants found a positive association between H. pylori infection and hypertension after adjusting for potential confounders. ^14^ By contrast, H. pylori status was not significantly different in patients with different grades of hypertension. ^15^ Given the above introduction and the diversity of the results about H. pylori infection and hypertension, the need to conduct a study to determine H. pylori infection with hypertension is quite clear. Thus, to further investigate the potential role of H. pylori infection in hypertension, a meta-analysis was conducted to provide a basis for intervention in hypertension.

## Methods

### Literature retrieval

All papers on the relationship between H. pylori and hypertension, published from 1996 to 2019, were selected in this meta-analysis. The articles were searched by titles and/or abstracts that contained ther terms “H. pylori” or “helicobacter pylori” and “hypertension” or “high blood pressure”, and which were published in Chinese on the Chinese Wanfang Data Knowledge Service Platform and in the Chinese National Knowledge Infrastructure (CNKI), and in English in the PubMed and Google Scholar Databases. Finally, the references were again filtered to avoid omission, in the screening process of reading the papers.

### Inclusion criteria and exclusion criteria

All studies that were identified by the literature search were selected according to the following essentials: patients (individuals who were diagnosed according to systemic arterial hypertension diagnosis standard); exposure (H. pylori infection); comparator (normotension); outcome (association between H. pylori infection and hypertension); and study design (cross-sectional or case-control study). Studies were excluded if they were ecological and cohort studies, if they were not grouped by hypertension and normotension, if the number of H. pylori patients in each group cannot be determined, or if there were possible errors. If the same studies duplicated in different databases or the study population overlapped, only the largest one was selected.

### Data extraction and quality assessment in the process

According to the purpose of this study, two independent researchers selected titles and/or abstracts to be included in the articles, and finally reached a consensus through a third expert’s evaluation. The required studies were read through the full articles, and the following information and characteristics were recorded: the first author’s name, year of publication, country, type of study, average age, number of participants, and testing for H. pylori infection.

## Statistical analysis

All statistical analyses were performed using Stata 12.0. Pooled odds ratios (ORs) with corresponding 95% confidence interval (CI) were considered as the effect size for all the eligible studies. Two methods (Cochran Q test and the I ^2^ statistic) were used to assess the statistical heterogeneity among the summary data: if p<0.05 was considered to be statistically significant for heterogeneity, and the I ^2^ statistic suggested a significant heterogeneity with a value of >50%. ^16^ A random-effects model was used to calculate the overall effect size estimate in this meta-analysis. To examine the sources of heterogeneity among the eligible studies, subgroup analyses were conducted according to different characteristics, such as study design (cross-sectional and case-control studies), study country (Asian and Western) and testing for H. pylori infection (urea breath test, serological test, among others). The urea breath test (UBT) included 13C-UBT and 14C-UBT; the serological test included the Colloidal gold method and ELISA; others included Giemsa staining and not available (N/A). Sensitivity analysis was performed to evaluate the effects of individual study on the summarized effects estimate and the stability of results. Begg’s and Egger’s regression tests were used to assess the publication bias. The meta-regression method was conducted for measurement data, such as sample size, average age, and gender ratio, and a two-tailed p-value <0.05 was defined as statistically significant.

## Results

### Basic characteristics of included articles ^17^^-^^33^

A flow diagram of studies for systematic review was shown in Figure 1 . Of these, 80 duplicate studies were excluded, and, based on the titles and abstracts of the remaining articles, 57 studies were also excluded. Ultimately, 17 publications were selected after examining the full text of these 22 publications, as specified in the flow diagram.

**Figure 1:** Flow diagram of studies included in the meta-analysis.

In total, six cross-sectional studies and eleven case-control studies were selected in the meta-analysis from 1996 to 2019. Among the 17,226 participants, the prevalence of H. pylori in 6,376 hypertensive patients was 64.9% and in 10,850 normotensives was 56.3%. The main characteristics of the studies included in this review were presented in Table 1 . ^17^^-^^33^

**Table 1:** Basic characteristic of the seventeen articles included herein

Studies	Year	Country	H. pylori ascertainment	Hypertension (H. pylori + / -)	Normotensive (H. pylori + / -)	Average age	Study design	Gender ratio *	p threshold
Yang ^17^	2016	China #	14C-UBT	78(66/12)	78(37/41)	N/A	Case-control study	0.79	0.05
Liu ^18^	2015	China #	14C-UBT	150(125/25)	150(71/79)	63.60	Case-control study	1.04	0.05
Huai ^19^	2014	China #	13C-UBT	535(338/197)	1051(232/819)	68.30	Cross-sectional study	2.68	0.05
Ma ^20^	2015	China #	Giemsa staining	112(42/70)	170(53/117)	N/A	Case-control study	N/A	0.05
Chu ^21^	2014	China #	IgG Colloidal gold	150(67/83)	50(14/36)	60.85	Case-control study	1.41	0.05
Hu ^22^	2017	China #	N/A	176(82/94)	568(224/344)	52.53	Cross-sectional study	1.23	0.05
Lip ^23^	1996	UK $	ELISA≥8 units/ml).	124(106/18)	38(25/13)	53.06	Case-control study	1.03	0.05
Liu ^24^	2007	China #	ELISA	488(189/299)	942(317/625)	46.56	Cross-sectional study	0.71	0.05
Sun ^25^	2018	China #	14C-UBT	90(76/14)	65(34/31)	44.74	Case-control study	1.12	0.05
Li ^26^	1999	China #	IgG ELISA	42(16/26)	60(18/42)	55.41	Case-control study	1.62	0.05
Zhao ^27^	2019	China #	14C-UBT	102(43/59)	102(18/84)	47.85	Case-control study	0.94	0.05
Migneco ^28^	2003	Italy $	13C-UBT	72(40/32)	70(35/35)	52.51	Case-control study	0.95	0.05
Vahdat ^29^	2013	Iran #	ELISA >30 RU/ml	459(316/143)	1295(764/531)	40.79	Cross-sectional study	0.97	0.05
Zheng ^30^	2014	China #	Giemsa staining	112(42/70)	170(53/117)	52.19	Case-control study	1.29	0.05
Sung ^31^	2003	Korea #	ELISA≥6 U/mL	2838(1964/874)	5509(3818/1691)	48.06	Cross-sectional study	1.36	0.05
Sotuneh ^32^	2014	Italy $	ELISA>20 ur/ml	808(606/202)	492(385/107)	69.23	Cross-sectional study	N/A	0.05
S.VSM ^33^	2012	India #	IgG ELISA>40 ur/ml	40(18/22)	40(9/31)	44.09	Case-control study	1.05	0.05

UBT, urea breath test; ELISA, enzyme linked immunosorbent assay; N/A, not available

* Gender ratio: male /female

# Asian countries

$ Occident countries.

### Results of meta-analysis

Based on the random effects model in Figure 2 , the general estimate of the pooled OR (95% CI) of H. pylori and hypertension was 2.07 (1.46–2.94), which was statistically significant (p < 0.001).

**Figure 2:** A forest plot of the association between H.pylori infection and hypertension.

### Subgroup analysis according to region, study design, and test method

For the subgroup analysis by region (Asian in 5,372 hypertensives and 10,250 normotensives, Western in 1,004 hypertensives and 600 normotensives), the H. pylori infection was associated with the risk of hypertension in Asian countries (OR 2.26, 95%CI 1.51-3.38; I^2^= 95.1%, p < 0.05), while no significant difference was found in Western nations.

In the subgroup analysis grouped by study design, the pooled OR for H. pylori infection for hypertension was 2.53 (95% CI 1.72-3.72; I^2^= 72.7%, p < 0.05) in the case-control studies. No statistical difference was observed in the cross-sectional studies ( Figure 3 and 4 ).

**Figure 3:** Forest plot of subgroup analyses comparing Asia with the West.

**Figure 4:** Forest plot of subgroup analyses comparing case-control study with cross-sectional study.

In the subgroup analysis of studies that used UBT to test H. pylori infection, H. pylori infection was associated with an increased risk for hypertension (OR 4.13, 95% CI 2.60-6.54; I^2^= 76.7%, p < 0.05). H. pylori infection was also associated with a high risk for hypertension in a subgroup analysis of studies in a serological test (OR 1.33, 95% CI 1.04–1.68; I^2^= 77.1%, p < 0.05) ( Figure 5 ).

**Figure 5:** Forest plot of subgroup analyses comparing in different diagnostic methods.

### Publication bias

Begg’s test was used to evaluate publication bias, using the funnel plot ( Figure 6 ). This was an asymmetry in the funnel plot for eligible studies. The significant publication bias was also identified in the Egger’s regression test (p = 0.047).

**Figure 6:** Begg’s Funnel plot with pseudo 95% confidence interval.

### Sensitivity analysis

A sensitivity analysis was performed to test the sources of heterogeneity and assess the stability of results. The sensitivity analysis results showed that no individual study had extreme influence on the pooled odds ratio ( Figure 7 ).

**Figure 7:** Sensitivity analyses for association between H. pylori and hypertension.

### Meta regression

Individual studies without an average age (n = 2) or without the number of men and women (n = 2) were excluded. Results of univariable meta-regressions show the lack of significant effects of sample size (p = 0.181), average age (p = 0.542), as well as gender ratio (p = 0.367) on the association between H. pylori infection and risk of hypertension in the studies (Supplementary Material: Supplementary Figures 1-3).

## Discussion

To the best of our knowledge, this is the first systematic review study to demonstrate that the relationship between H. pylori infection and hypertension. The finding showed that the prevalence of H. pylori infection was positively associated with hypertension, and this result was consistent in the case-control group in the subgroup analysis. Mendel et al. ^34^ first put forward the hypothesis of the relationship between H. pylori infection and coronary heart disease. Subsequently, some scholars have carried out research on H. pylori infection in cardiovascular disease. ^35^

A previous cohort study demonstrated that H. pylori-infected, chronic active gastritis positive individuals showed a 29% higher risk for hypertension development during the follow-up period. ^36^ One Chinese study indicated that H. pylori infection was independently associated with higher DBP but not SBP after adjusting for covariates. ^14^ However, several studies showed that H. pylori infection did not influence blood pressure. ^37^^,^^38^ Of course, we cannot conclude that H. pylori infection facilitates hypertension; however, our meta-analysis now supports the association between H. pylori infection and hypertension.

The prevalence of H. pylori infection varies markedly in different countries and regions. H. pylori infections in Asian nations were common and extensively distributed, and the average infection rate of H. pylori infection in China was 58.07%, with 50% in the age group of 10 to 20 years. ^39^ Epidemiological investigation showed that the cytotoxin-associated gene A (CagA) positive strain from different geographic areas exhibits clear phylogeographic differentiation. More than 90% of H. pylori isolates from East Asian countries, such as China and Japan, contain CagA protein, while only 60% - 70% of H. pylori isolates from Western countries, such as the United States, contain the CagA protein. ^40^ Moreover, Migneco A et al. ^28^ found that only among the high CagA positive patients did the DBP decrease, most visibly after the H. pylori had been eradicated, and may be related to the molecular relationship between the CagA antigen of H. pylori and some peptides expressed by endothelial cells and smooth muscle cells. It is notable that the OR of hypertension in Asian populations with H. pylori infection was 2.26-fold more than that of the normotensive subjects in our study. These results suggest that ethnic origin may have a potential impact on the relationship between H. pylori infection and hypertension.

Another issue in determining the relationship between H. pylori infection and hypertension is the method used to test for H. pylori infection. Although serological testing is a popular screen for H. pylori infection, it does not guarantee current H. pylori infection. ^41^ Diagnostic accuracy of using UBT to detect H. pylori infection in the Asian population, especially 13C-UBT, which had outstanding diagnostic accuracy with a sensitivity of 97% and a specificity of 96%. ^42^ In our subgroup analysis, the risk for hypertension was higher when H. pylori infection was determined using UBT compared with other tests.

The mechanisms that link H. pylori infection to hypertension remain unclear. There are several hypotheses supporting the relationship, and one of the most plausible is the levels of inflammatory cytokines. Epstein et al. ^43^ indicated that the chronic inflammation caused by H. pylori infection may consequently result in advanced atherosclerosis. In addition, a prospective study has demonstrated that H. pylori had a positive association with high LDL and low HDL. ^44^ Lipids, as an integral part of the cell membrane, play an important role in the development of hypertension. ^45^ It is speculated that H. pylori infection leads to the abnormal metabolism of LDL-C, HDL-C, and TC, which in turn results in hypertension. H. pylori infection destroys immune tolerance and causes autoimmune reaction, which may participate in the pathogenesis of hypertension. ^46^^,^^47^ Age were found to be independent predictors of H. pylori infection, and an increasing trend of prevalence with age ,while no difference in prevalence was found between both sexes. ^48^ Furthermore, men were more than twice as likely to develop cardiovascular disease as women at least under the age of 60. ^49^ However, our study indicates a lack of significant effects of age and gender on the association between H. pylori infection and the risk of hypertension. It is possible that different ethnicity, assessment of H. pylori infection, sample sizes, and potential confounders may contribute to the discrepancies.

### Limitations

There are several limitations in our study. First, all included studies were observational, which made it difficult to estimate a causal association. Second, significant heterogeneity was identified in the meta-analyses, and it was not possible to adjust for potentially confounding variables by other inaccessible information. Third, most original studies were performed in the Asian population, and a publication bias was found in the meta-analysis.

## Conclusions

In conclusion, our results indicated that H. pylori infection is positively associated with hypertension. The strategies of preventing H. pylori infection and eradicating H. pylori may have a significant impact on the prevention and treatment of hypertension, and warrants further evaluation.
